# Variability in the Practice of Fertility Preservation for Patients with Cancer

**DOI:** 10.1371/journal.pone.0127335

**Published:** 2015-05-26

**Authors:** Kasey A. Reynolds, Natalia M. Grindler, Julie S. Rhee, Amber R. Cooper, Valerie S. Ratts, Kenneth R. Carson, Emily S. Jungheim

**Affiliations:** 1 Division of Reproductive Endocrinology, Department of Obstetrics and Gynecology, Washington University School of Medicine, St. Louis, Missouri, United States of America; 2 Division of Public Health Sciences, Washington University School of Medicine, St. Louis, Missouri, United States of America; 3 Division of Oncology, Washington University School of Medicine, St. Louis, Missouri, United States of America; 4 Research Service, St. Louis Veterans Affairs Medical Center, St. Louis, Missouri, United States of America; University Hospital of Münster, GERMANY

## Abstract

Fertility is important to women and men with cancer. While options for fertility preservation (FP) are available, knowledge regarding the medical application of FP is lacking. Therefore we examined FP practices for cancer patients among reproductive endocrinologists (REs). A 36 item survey was sent to board-certified REs. 98% of respondents reported counseling women with cancer about FP options. Oocyte and embryo cryopreservation were universally offered by these providers, but variability was noted in reported management of these cases—particularly for women with breast cancer. 86% of the respondents reported using letrozole during controlled ovarian stimulation (COS) in patients with estrogen receptor positive (ER+) breast cancer to minimize patient exposure to estrogen. 49% of respondents who reported using letrozole in COS for patients with ER+ breast cancer reported that they would also use letrozole in COS for women with ER negative breast cancer. Variability was also noted in the management of FP for men with cancer. 83% of participants reported counseling men about sperm banking with 22% recommending against banking for men previously exposed to chemotherapy. Overall, 79% of respondents reported knowledge of American Society for Clinical Oncology FP guidelines—knowledge that was associated with providers offering gonadal tissue cryopreservation (RR 1.82, 95% CI 1.14–2.90). These findings demonstrate that RE management of FP in cancer patients varies. Although some variability may be dictated by local resources, standardization of FP practices and communication with treating oncologists may help ensure consistent recommendations and outcomes for patients seeking FP.

## Introduction

Future fertility is important to young women and men diagnosed with cancer [1–3]. Awareness of fertility preservation (FP) options has improved over recent years, and more patients are being referred by their oncologists to discuss FP options with reproductive specialists [4–7]. Most FP options require the application of Artificial Reproductive Technologies (ARTs)—established technologies most commonly used to treat infertility patients [8]. ARTs include oocyte and embryo cryopreservation for women, and intracytoplasmic sperm injection of oocytes using cryopreserved spermatozoa for men (Table 1). Experimental FP options requiring testicular or ovarian tissue cryopreservation are available for prepubertal patients and for women who lack the time required for established FP methods [1,9,10].

**Table 1:** Standard and experimental options utilizing ART for FP in cancer patients [1,9,10].

**ART FP options for women**	
Oocyte banking	Established
Embryo banking	Established
Ovarian tissue cryopreservation	Experimental
**ART FP options for men**	
Sperm banking with in vitro fertilization and intracytoplasmic sperm injection	Established
Testicular tissue cryopreservation	Experimental

Both experimental and established FP services are provided by Reproductive Endocrinologists—physicians trained in both Obstetrics and Gynecology and in Reproductive Endocrinology, and collaborating urologists. RE experience in providing FP services to patients with cancer is growing, but as recently pointed out in the 2013 update to the ASCO guidelines for fertility preservation for patients with cancer, there is a paucity of well-designed studies and outcome data focused on the application, success, and effects of FP on patients with cancer [1].

**Fig 1:**
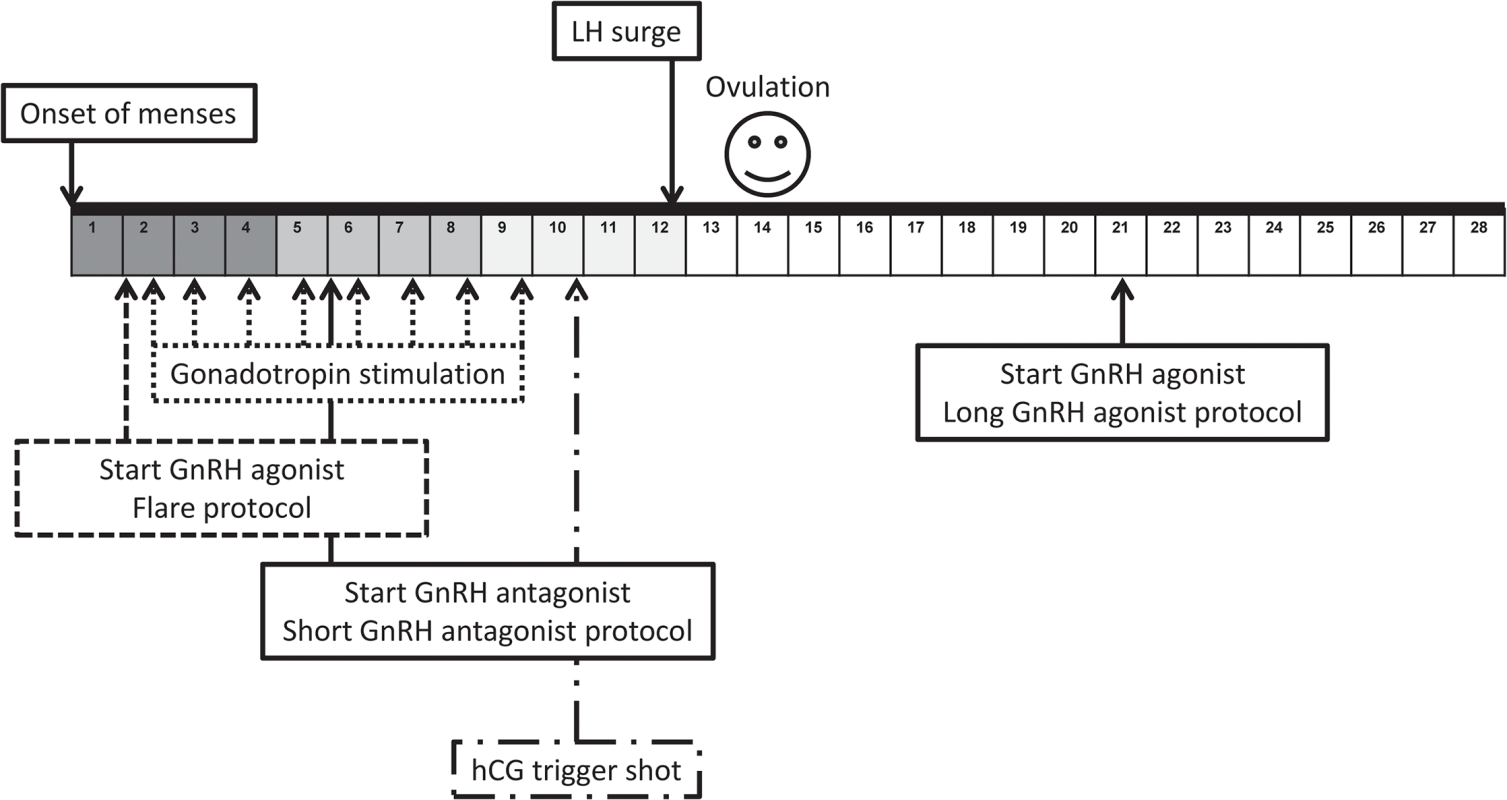
Standard COS protocols and timing in menstrual cycle. Events depicted above the grid occur in natural menstrual cycles whereas events depicted below the grid depict timing of administration of medications for COS protocols. Color in the grid is representative of rising estradiol levels during the menstrual cycle and COS protocols.

Given the lack of data available for FP outcomes in patients with cancer, concerns have been raised over the application of conventional ART practices in cancer patients [3,11]. Many of these concerns focus on the gonadotropin stimulation regimens used to prepare the ovaries for oocyte harvest—a treatment commonly referred to as controlled ovarian stimulation (COS). Traditional COS regimens have centered around normal physiologic events of the menstrual cycle to stimulate recruitment of multiple oocyte-containing, ovarian follicles. This process requires appropriate timing with the menstrual cycle and it associated elevations in serum estradiol levels (Fig 1) [8]. COS is generally well tolerated, but there are associated risks that may be particularly undesirable for women with cancer [3]. For instance, there is a theoretical risk that the supraphysiologic estrogen levels resulting from COS could stimulate the growth of estrogen-sensitive tumors or increase the risk of cancer recurrence [3]. Women undergoing COS are also at risk for ovarian hyperstimulation syndrome (OHSS) and thromboembolic events which could delay or complicate planned cancer treatments [11]. Furthermore, for women who need to initiate cancer treatment quickly the time required for traditional COS regimens can be prohibitive. Concerns over such risks have led physician-scientists to propose modifications in traditional COS protocols including the incorporation of letrozole into COS for women with breast cancer to curb rising estradiol levels, and initiating COS randomly in the menstrual cycle to speed up the time to oocyte harvest for women with little time available to delay cancer treatment (Fig 1) [1,12–15].

Whether or not modifications for cancer patients undergoing FP procedures have been assimilated into FP practice is unknown. We conducted a national survey of REs to clarify deficiencies and knowledge gaps in current FP practice for patients with cancer and inform improvements in future practice.

## Materials and Methods

### Survey Development and Content

We developed a questionnaire that was piloted among RE’s at the Midwest Reproductive Symposium meeting held in Chicago, Illinois, in May 2012. The survey focused on three areas: 1) Provider demographics; 2) Fertility preservation for women with cancer; and 3) Fertility preservation for men with cancer. Several questions regarding contraceptive counseling were also asked. The final survey consisted of 36 questions (S1 Appendix).

### Survey Distribution

This cross-sectional survey was approved by the Human Research Protection Office, the institutional review board (IRB) at Washington University. Per our IRB, respondents provided implied consent by responding to the survey. We used the Society for Reproductive Endocrinology and Infertility (SREI) membership directory (www.socrei.org) to identify participants. SREI represents fellowship-trained Reproductive Endocrinologists who are board-certified in both Obstetrics and Gynecology and the subspecialty of Reproductive Endocrinology by the American Board of Obstetrics and Gynecology. 791 members were identified. We excluded members without email addresses (n = 5), members practicing outside the United States (n = 20), and members from our own practice (n = 7). 759 e-mail invitations containing a link to the anonymous web-based survey were sent on August 3, 2012. Three reminders were sent between August and October, 2012. Participants had 8 weeks to complete the survey.

### Data Analysis

The survey was implemented using SurveyMonkey (Portland, OR). Results were transferred to SPSS version 20 (Armonk, NY) for analysis. Frequencies and proportions were summarized for physician demographics and practices. Relative risks (RR) with 95% confidence intervals were estimated to compare knowledge of ASCO guidelines and practices.

## Results

The response rate was 13% (88/684). 51 e-mail addresses were undeliverable, and auto-responses indicated 24 addressees were unavailable. 596 invitations were unanswered.

### Provider Demographics

Table 2 summarizes respondent demographics. 51% were male, and 49% were female. The average age of survey participants was 46 years, and participants represented both academic (61%) and private (39%) practices. Overall, 98% of respondents reported offering FP services for patients with cancer. Of these, 78% reported an affiliation with a cancer center and 71% reported familiarity with ASCO guidelines for fertility preservation in patients with cancer. Respondents came from 28 different states.

**Table 2:** Respondent demographics.

Characteristic		N = 89
Gender	Male	49% (44)
	Female	47% (42)
	Chose not to answer	3% (3)
Age (mean years ± SD)		48.8 ± 11.8
Practice Setting	Private Practice	38% (34)
	Academic	60% (54)
	Chose not to answer	1% (1)
Associated with a cancer center (n)		78% (65)
Knowledge of ASCO FP guidelines (n)		78% (65)

### Fertility Preservation for Women with Cancer: Controlled Ovarian Stimulation

Physicians’ responses varied regarding COS preference for FP cases in women with cancer. Regarding how to dose gonadotropins for COS, most physicians would either use a standard amount of gonadotropin (45%) or higher doses (45%), with only 10% stating that they would use a gentler stimulation protocol with lower doses of gonadotropins for FP patients with cancer. Survey respondents also differed in their preferred COS protocol for FP. Most (80%) preferred a GnRH agonist-based stimulation protocol (a protocol associated with a longer stimulation time than other options [11]), while others preferred a GnRH antagonist-based protocol (8%). The remainder did not have a preference, stating they tailored the protocol to the patient (12%).

Regarding preferred agent for inducing oocyte maturation in COS, 80% of respondents preferred a standard hCG trigger, and 20% preferred GnRH agonist trigger (a modification to traditional protocols that has been proposed to reduce the risk of ovarian hyperstimulation syndrome in women with cancer undergoing COS for FP [11,16,17]). This finding was in line with stimulation protocol preferences, as hCG is used concurrently with a GnRH agonist stimulation regimen, and a GnRH agonist trigger can be utilized in GnRH antagonist cycles.

Another aspect of stimulation in which survey respondents disagreed was whether or not to start COS randomly (e.g. not early in the follicular phase). Approximately half (48%) of surveyed physicians stated that they would randomly start COS for FP patients, while the remainder, 52%, would not (random starts are modifications to traditional protocols to speed the time to oocyte retrieval [11,18]).

Ultimately, 40% of physicians would cancel FP COS if the patient had poor ovarian response to gonadotropin—commonly defined as fewer than 3 developing ovarian follicles. The remaining 60% would proceed to retrieval regardless of the number of developing follicles. Many respondents commented that FP patients often only have “one chance” at stimulation, so cancellation was not an option.

Several survey questions focused on the management of COS for oocyte and embryo cryopreservation in patients with breast cancer—specifically on the use of letrozole to blunt estrogen exposure during COS. 86% of the respondents reported using letrozole during COS in patients with estrogen receptor positive (ER+) breast cancer to minimize patient exposure to estrogen. 20% of respondents stated they had used tamoxifen as an adjuvant to COS in ER+ breast cancer patients as well. Comments from those reporting they used letrozole in COS for women with ER+ breast cancer included the following: “Yes, to reduce estrogen as used in published data,” “Based on literature and recommendation of oncologist,” “Most published experience in the literature,” “To diminish estradiol production based on Oktay protocol; reassures patients and oncologists who are nervous with supraphysiologic estradiol levels.” One respondent stated they would not offer COS at all to women with ER+ cancer. Comments from the 14% who disagreed with using letrozole in COS for women with ER+ breast cancer included the following: “Not much change in estradiol, estradiol levels much higher in pregnancy anyway,” “Show me the evidence!,” “Oncologists at our center don't feel that there is any advantage for short term.”

49% of respondents who reported using letrozole in COS for patients with ER+ breast cancer reported that they would also use letrozole in COS for women with ER negative breast cancer. Comments from these respondents included the following: “Try to limit estradiol exposure in any breast cancer,” “Estradiol has activity besides through classic receptor mechanism,” “Oncologist still prefers that we do”, and “Due to limited safety data for process.” Some respondents stated they (20%) also had used tamoxifen when stimulating ER+ breast cancer patients.

### Fertility Preservation for Women with Cancer: Ovarian Tissue Cryopreservation

20% of survey respondents reported offering ovarian tissue cryopreservation as an FP option. Interestingly, 100% of the survey participants who offer ovarian tissue cryopreservation were also familiar with ASCO guidelines, while none of the physicians who were unfamiliar with ASCO guidelines offer the option of ovarian tissue cryopreservation (16/63 vs. 0/17).

### Fertility Preservation for Men

83% of respondents reported offering semen cryopreservation services to male patients with cancer. 22% of those offering sperm banking responded that they do not recommend banking to men with cancer who have already been exposed to chemotherapy. 33% reported offering electroejaculation services, and 84% reported offering emergent testicular sperm extraction (TESE)—options for men who are unable to provide a semen specimen through masturbation. A larger proportion of REs who were familiar with ASCO guidelines offered TESE (RR 1.61, 95% CI 1.04–2.50). This result combined with those offering OTC revealed that overall those who offered gonadal cryopreservation were more likely to also be familiar with ASCO guidelines (1.82, 95% CI 1.14–2.90).

### Contraceptive Counseling

83% of respondents reported advising female patients with cancer to contracept during chemotherapy while 59% of respondents reported counseling men to contracept during chemotherapy. Comments associated with responses included the following: “I leave this to the med oncologists,” “discussion usually left to others on the team,” “doesn’t come up in our consults,” “I don’t, but I should.”

## Discussion

To our knowledge this is the first survey of REs on practice patterns, protocols, and preferences in providing FP services for people with cancer. Our results highlight a lack of consistency in FP practice—specifically in management of COS protocols for women with cancer, recommendations regarding banking of sperm after initiation of chemotherapy for men, and counseling regarding contraception for both men and women with cancer. We believe this latter point is particularly important in light of the fact that while we emphasize that fertility is at risk with many cancer treatments, pregnancy is still possible. The FP consultation is an ideal time to discuss contraceptive options—especially in young breast cancer patients who often stop oral contraceptive pills after diagnosis. We also believe the lack of consensus in responses amongst providers is consistent with the recent update to the ASCO guidelines for fertility preservation in patients with cancer which states, “fertility preservation methods are still applied relatively infrequently in patients with cancer, limiting greater knowledge about the success and effects of different interventions” [1]. Thus improvements in provider collaboration are required to optimize treatment outcomes and survivorship for young patients with cancer.

Our results highlight a variety of opinions and preferences among REs regarding COS in women for oocyte and embryo cryopreservation. 90% of responding physicians reported they would use standard (45%) or higher doses of gonadotropins for COS (45%). Previous work has demonstrated diminished ovarian reserve in women with cancer which may require more gonadotropin. On the other hand there are concerns that the supraphysiologic levels of estrogen associated with COS could result in OHSS or increase the risk for thromboembolism [1,11]. Given this risk, it is not surprising that some of the respondents stated that they would use lower doses of gonadotropin (10%). Which approach is best is unknown.

Our results also show disagreement among REs regarding the use of letrozole or other estrogen-opposing agents in COS for women with breast cancer. The only safety data on COS in women with breast cancer is from a 2008 study by Azim et al. from JCO. In this study, women with breast cancer pursuing FP with oocyte or embryo cryopreservation were placed on a GnRH antagonist-based COS protocol incorporating letrozole to keep circulating estradiol levels low [13]. There was no difference in disease recurrence between the 79 women who underwent COS versus the 136 women who did not with 23 months of follow up in the COS group and 33 months in the control group. In an editorial response to the study concerns were raised over the small study size, limited follow up, and lack of randomization. In the editorial, Dr. Ann Partridge cautioned that, “Legitimate concern remains that the ovarian stimulation could have a negative impact on outcome. More extended follow-up is critical, particularly for women with hormone receptor-positive disease who have a risk of disease recurrence that extends for many years.” On the other hand, Partridge acknowledged, “decisions in the clinic cannot always wait until more solid data are available.” Given that Dr. Azim’s data using GnRH antagonist-based COS is the only data available regarding safety of COS in women with cancer, it is surprising to note that only 12% of the respondents in this study reported that they preferred GnRH antagonist-based COS for women with cancer.

Several groups have published reviews to help facilitate management of FP in patients with cancer. These organizations include the International Society for Fertility Preservation[19], fertiPROTEKT—a collaboration of centers in Germany, Switzerland and Austria [17], and individual centers with an abundance of experience in providing FP for cancer patient [11,20]. All of these documents provide guidance, but again outcome is lacking.

According to our survey, most REs are familiar with ASCO guidelines for fertility preservation in cancer patients. While these guidelines were not focused on Reproductive Endocrinologists, we believe the responses from our survey highlight the fact that the ASCO guidelines are a source document for those providing FP services to patients with cancer. Statements regarding the use of agents like letrozole as COS adjuvants for breast cancer patients and options for contraception in cancer patients may be appropriately placed in the oncology literature through such documents or other supplements. Our data indicate that REs who are familiar with the ASCO guidelines are more likely to offer experimental FP options like gonadal tissue cryopreservation. Given this, it is possible the responding providers may be more abreast of the FP literature for cancer patients. Again we believe that the ASCO guidelines may be a forum for REs and clinicians providing cancer care alike.

Perhaps one of the best contributions our study provides is the commentary provided by practicing REs. Conflicting statements regarding the use of letrozole in ER+ breast cancer patients in particular draws attention to the fact that some consensus with input from oncologists would be timely and that follow up outcome data on patients with cancer who pursue FP is needed to ensure consistent and safe outcomes in the future[3].

There are several important limitations to our study. Our response rate was low (13%). On the other hand, this response rate is consistent with surveys of Oncologists regarding FP counseling and referral patterns[6,21–23]. In surveys of clinicians providing oncology care, response rates ranged from 7% to 12.5% among physicians[21,22], and up to 22% among clinical nurses[22]. A 32% response rate was achieved in a survey of Oncologists published in JCO in 2008 but clinicians were paid a $100 honorarium[6].

Because of our response rate, we cannot be certain our results are truly representative of RE practice patterns. However, there was a representative sampling from both private practice and academic centers across the country. Importantly, the physicians who responded to the survey represent states in which the bulk of IVF takes place nationally (California, Florida, Illinois, New Jersey, New York, and Texas)[24]. States not represented in this survey included: Arkansas, Alaska, Delaware, Idaho, Iowa, Kansas, Kentucky, Maine, Minnesota, Mississippi, Nebraska, Nevada, New Hampshire, New Mexico, North Dakota, Oregon, South Dakota, Utah, Vermont, West Virginia, Wisconsin, Wyoming. Of these states, there are no ART centers registered with Society for Assisted Reproductive Technologies in Alaska, Maine or Wyoming. Among the others, in the most recent data provided by SART 12,269 cycles of ART were performed of 154,412 reported nationwide—just under 8%[25]. Given this we contend that our survey is likely representative of the majority of physicians practicing FP.

Our response rate may be secondary to repeat requests of this group to respond to surveys, or survey fatigue [26]. We surveyed SREI members and members of the Society for Reproductive Surgeons less than one year prior to this survey achieving a 43% response rate [27]. We cannot be certain that the people who responded to the current survey responded to the previous survey, but we doubt many people who failed to respond to our previous survey responded to the current survey. For these reasons we estimate 25% (88/370) of eligible participants who responded to our previous survey responded to the current survey. Also our previous survey inquired about a general topic in reproductive medicine. There could be less interest or decreased level of comfort among those surveyed in the subject of FP—we suspect this is the case. Along these same lines, it is possible that only RE specialists routinely providing FP care who are abreast with the FP literature responded to our survey, thus it is possible FP practices vary more among RE specialists who did not respond. There is also potential reporting bias in our results as the responses are based on physician recall, rather than actual practice data.

Our data suggests that FP practice varies and it highlights important areas where attention is needed. We believe this study is a particularly important addition to the literature as FP practice patterns amongst REs for patients with cancer have gone unreported and REs are responsible for directly applying FP options. Consistency in future practice is important as the cancer population served by FP is young and focused on survival. Consistency in practice will ensure their oocytes, embryos, sperm and gonadal tissue is handled appropriately for optimal fertility treatment outcomes. Consistency will only come with collaboration and continued communication among clinicians providing FP services and Oncologists.

## Supporting Information

S1 Appendix Final 36-item survey. (DOCX)
