# Supplementary material for: Variability in the Practice of Fertility Preservation for Patients with Cancer
Source: PLoS One. 2015 May 26;10(5):e0127335. doi: 10.1371/journal.pone.0127335 (PMC4444257; doi:10.1371/journal.pone.0127335)
Supplement: S1 Appendix — (DOCX) [file pone.0127335.s001.docx]

Fertility Preservation Physician Practice Patterns Survey

1. How would you describe your practice?
   1. University/Academic Institution
   2. Private Practice affiliated with an academic institution
   3. Private Practice
   4. Other
2. Are you familiar with the American Society of Clinical Oncology (ASCO) guidelines regarding fertility preservation?

1. Does your practice offer fertility preservation services for cancer patients and other patients facing gonadotoxic therapies? If not, please skip ahead to page 3.
2. Does your practice have a working relationship with a cancer center?
3. For the purposes of fertility preservation for women facing gonadotoxic treatments, approximately how many OOCYTE banking cases do you perform per year?
4. For the purposes of fertility preservation for women facing gonadotoxic treatments, approximately how many EMBRYO banking cases do you perform per year?
5. Do you have any restrictions for offering fertility preservation services and oocyte/embryo banking in the setting of GONADOTOXIC THERAPIES?
   1. If you answered "yes" to the above question, what variables and parameters do you use to establish a cutoff?
6. In your protocol for women facing GONADOTOXIC THERAPY who are undergoing oocyte/embryo banking for fertility preservation, how do you normally dose gonadotropins?
   1. more aggressive protocol - a higher dosage of gonadotropins than I would normally use
   2. less aggressive protocol - a lower dose of gonadotropins than I would normally use
   3. standard protocol – no modifications
   4. other – please specify
7. Which of the following do you utilize when determining your gonadotropin starting dose for women undergoing COH for fertility preservation?
   1. AMH, what value?
   2. Basal FSH, what value?
   3. Age, what value?
   4. Antral follicle count, what value?
   5. Other, what value?
8. For women facing GONADOTOXIC TREATMENTS undergoing oocyte/embryo banking for fertility preservation, which of the following stimulation protocols do you prefer?
   1. GnRH agonist
   2. GnRH antagonist
   3. no preference
   4. Other (please specify)
9. When utilizing a GnRH antagonist protocol for fertility preservation patients facing GONADOTOXIC THERAPIES, which is your PREFERRED AGENT for inducing follicular maturation?
   1. GnRH agonist
   2. hCG (recombinant)
   3. hCG (urinary)
   4. Other (please specify)
10. Do you routinely administer letrozole with your controlled ovarian hyperstimulation protocol for ER positive breast cancer patients?
11. Do you routinely administer letrozole with your controlled ovarian hyperstimulation protocol for ER NEGATIVE breast cancer patients as well?
12. Do you use tamoxifen during controlled ovarian hyperstimulation with your breast cancer patients undergoing fertility preservation?
13. Do you have a minimum number of follicles you will go to retrieval for in fertiltiy preservation patients?
14. Do you counsel female patients to contracept while receiving chemotherapy?
15. Do you ever begin gonadotropin for controlled ovarian hyperstimulation in the luteal phase for patients undergoing ovarian stimulation for fertility preservation?
16. Does your center offer OVARIAN TISSUE CRYOPRESERVATION for women facing potentially gonadotoxic treatments? If not, please skip to the next page.
17. Approximately how many cases of ovarian tissue cryopreservation has your center performed
18. Have you, or anyone in your practice, transferred ovarian tissue back to a patient yet/performed orthotopic transfer of this tissue?
19. If your center has performed orthotopic transfer of ovarian tissue, how many cases have been performed?
20. Does your center offer sperm cryopreservation services for these men?
21. Does your center offer electroejaculation for these men who cannot provide a banking sample by masturbation?
22. Does your center offer emergent TESE procedures and subsequent banking for these men who cannot provide a semen specimen by masturbation or electroejaculation?
23. Do you offer banking services for male patients who have ALREADY STARTED gonadotoxic chemotherapy and/or radiation therapy?
24. Do you counsel male patients to contracept during gonadotoxic therapy (i.e. chemotherapy or immunotherapy)?
